# Whole Genome sequencing data of *Pseudomonas aeruginosa* strain PA-Mosul-25, harboring the *bla*_VEB-9_ ESBL gene, isolated from a hospital environment in Nineveh, Iraq

**DOI:** 10.1016/j.dib.2026.112780

**Published:** 2026-04-16

**Authors:** Hiba K. Saeed, Talal S. Salih

**Affiliations:** Department of Medical Physics, College of Science, University of Mosul, Iraq

**Keywords:** *Pseudomonas aeruginosa*, Genome sequence, Sequence type 357, Antibiotic resistance genes, blaVEB-9 gene, Pathogenicity

## Abstract

In this study, we report the draft genome sequence of *P. aeruginosa* strain PA-Mosul-25, belonging to sequence type 357 (ST357), isolated from a hospital environmental surface in Nineveh, Iraq. The strain was isolated from a swab taken from the floor using a sterile cotton swab. The isolate was cultured on Pseudomonas Agar Base, which is a selective medium, followed by identification using the VITEK 2 system. The whole-genome sequencing was conducted using the Illumina NovaSeq X platform. The assembled genome has a total size of 6157,064 bp, organized into 124 contigs, with an N50 value of 126,162 bp and a GC content of 66.71 %. Genome annotation predicted 5673 protein-coding sequences, 53 tRNA genes, and 3 rRNA operons. Multilocus sequence typing classified the isolate as ST357. Genome analysis identified four β-lactam resistance genes including *bla*_VEB-9_, *bla*_OXA-10_, *bla*_OXA-50_, and *bla*_PAO_, and nine additional antimicrobial resistance genes including *aac*(6′)-Il, *aad*A1, *sul*1, *dfr*B2, *fos*A, t*et*(A), *cat*B7, *Oqx*B, and *qac*E. Comparative analysis showed high genomic similarity to P. aeruginosa WH-SGI-V-07,060 based on ANI and dDDH values. The genome sequence data of *P. aeruginosa* PA-Mosul-25 have been deposited in the National Center for Biotechnology Information under accession number JBUYZG000000000.1. These data provide a resource for further comparative genomic and antimicrobial resistance studies of ST357 isolates.

Specifications Table

**Table:** 

Subject	Biology
Specific subject area	Genomics, Molecular Microbiology, Bacteriology.
Type of data	Whole Genome Sequence Data in FASTA format, Analyzed, Tables, figures, deposited.
Data collection	Genomic DNA of Pseudomonas aeruginosa PA-Mosul-25 was extracted from a 24-h culture grown on Nutrient Agar using the Presto™ Mini gDNA Bacteria Kit. The DNA library was prepared using the TruSeq Nano DNA Library kit and sequenced on the Illumina NovaSeq X platform (Illumina, USA). Raw sequencing reads were quality-trimmed using Trimmomatic v0.30, and the genome was *de novo* assembled with SPAdes v4.2. Assembly statistics were generated using QUAST v5.3.0, while genome completeness and contamination were evaluated using CheckM v1.2.4. Genome annotation was performed using the Rapid Annotation using Subsystems Technology (RAST) server and the NCBI Prokaryotic Genome Annotation Pipeline (PGAP) v6.10. Comparative genome analysis included calculation of the Z-score using JSpecies v5.0.3. Digital DNA–DNA hybridization (dDDH) was estimated using the Genome-to-Genome Distance Calculator (GGDC) v3.0 web server, and average nucleotide identity (ANI) was determined using the Proksee server. Multilocus sequence typing (MLST) and allele profiling were performed using the PubMLST database. Antimicrobial resistance genes were predicted using the Comprehensive Antibiotic Resistance Database (CARD v4.0.1) and ResFinder v4.7.2. The pathogenic potential of the isolate was predicted using PathogenFinder v2.
Data source location	*P. aeruginosa* PA-Mosul-25 was isolated from a hospital environmental sample in Nineveh, Iraq, located at 36.3456° N latitude, 43.1575° E longitude.
Data accessibility	The draft genome sequence of *P. aeruginosa* PA-Mosul-25 is available in the NCBI GenBank database. Data identification number: Genome accession: JBUYZG000000000.1, BioProject accession: PRJNA1422713, BioSample accession: SAMN55318257, SRA: SRX32359117 Direct URL to data: https://www.ncbi.nlm.nih.gov/nuccore/JBUYZG000000000 https://www.ncbi.nlm.nih.gov/bioproject/PRJNA1422713 https://www.ncbi.nlm.nih.gov/biosample/SAMN55318257 https://www.ncbi.nlm.nih.gov/sra/PRJNA1422713
Related research article	None*.*

## 1 Value of the Data

• The dataset includes the genome sequence of *Pseudomonas aeruginosa* sequence type ST357 (ST357), an environmental isolate obtained from a hospital surface in Nineveh, Iraq, which provides comprehensive genomic information that was sequenced through next-generation sequencing technology.
• The results of the genome analysis show the presence of different antimicrobial resistance determinants, particularly β-lactam-associated genes, helping researchers to get more insights into the possible resistance profile of this *P. aeruginosa* strain isolated from the environment.
• The genomic sequence of the *P. aeruginosa* sequence ST357, known for its multidrug-resistant, is essential for further comparative genomic analyses and the study of the global spread and evolution of this lineage.
• This new dataset will contribute significantly to the genomic information deposited in the public database of the National Center for Biotechnology Information. Moreover, it will also offer valuable information about the epidemiology of these strains of *P. aeruginosa*, not only locally but also globally.

## 2 Background

*Pseudomonas aeruginosa* is a Gram-negative, aerobic, non-spore-forming bacterium characterized by polar flagella-mediated motility. It is a major opportunistic pathogen and a leading cause of healthcare-associated infections worldwide, contributing substantially to morbidity and mortality [1]. In addition to multidrug-resistant (MDR) isolates of *P. aeruginosa*, extensively drug-resistant (XDR), pandrug-resistant (PDR), and difficult-to-treat resistance (DTR) have emerged and disseminated worldwide [2,3]. The genome size of P. aeruginosa strains ranges from 5.5 to 7 million base pairs (Mb) [4], making it one of the largest genomes among bacterial pathogens that infect humans. This relatively large genome harbors a diverse array of genetic determinants, including antimicrobial resistance genes, virulence factors, and biofilm formation capabilities, all of which contribute to its pathogenicity and multidrug resistance [5]. The high risk clone ST357 of *P. aeruginosa* is of special concern because of its global dissemination, pathogenicity, and presence of various antibiotic resistance genes including extended-spectrum β- lactamases (ESBLs) in its genome [6]. Here, we present the draft genome sequence analysis of *P. aeruginosa* ST357 harboring the *bla*_VEB-9_ gene, designed as strain PA-Mosul-25 strain. This strain was isolated from an environmental sample collected at Al-Khansa governmental hospital in Nineveh, Iraq. We further assessed its genome features, performed pairwise genome analysis, determined its sequence type, and investigated the presence of antibiotic resistance genes.

## 3 Data Description

**Fig. 1:**
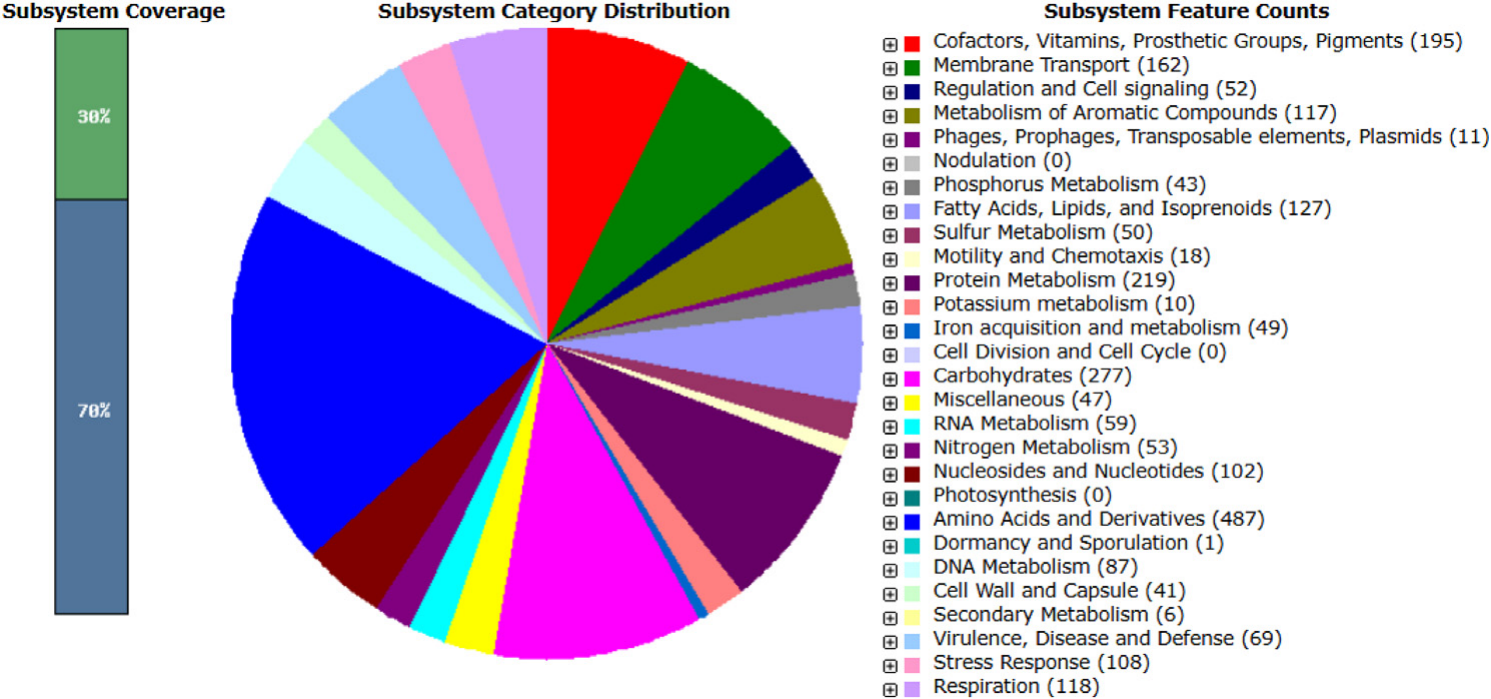
Distribution of subsystem categories in the genome of *P. aeruginosa* PA-Mosul-25.

**Fig. 2:**
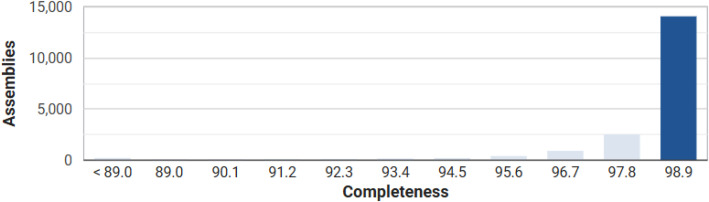
Genome completeness of *P. aeruginosa* PA-mosul-25 assessed using CheckM v1.2.4 with the prokaryotic genome annotation pipeline (PGAP) gene set and the *Pseudomonas aeruginosa-*specific CheckM marker set.

The draft genome sequence of *P. aeruginosa* strain PA-Mosul-25 has a total size of 6157,064 bp, and is composed of 124 contigs (>1145). The assembly shows an N50 value of 126,162 bp and a GC content of 66.71 %. A summary of the genome assembly and annotation statistics is presented in Table 1. Genome annotation predicted 5794 genes, including 5673 protein-coding sequences (CDSs), 53 tRNA genes, 3 rRNA genes, 4 ncRNA genes and 397 functional subsystems. Among these subsystem, genes involved in amino acids and their metabolism (487 genes) were the most abundant, followed by carbohydrate metabolism (277 genes), protein metabolism (219 genes), cofactors, vitamins, prosthetic groups and pigments (195 genes) and membrane transport (162 genes). The distribution of genes across the subsystem categories is illustrated in Fig. 1. Quality assessment of the genome assembly using CheckM indicated a genome completeness of 98.79 % with an estimated contamination level of 1.15 % (Fig. 2).

**Table 1:** General genome characteristics of *P. aeruginosa* PA-Mosul-25*.*

Features	Value
Total length size (bp)	6157,064
DNA GC content (%)	66.71
Genome coverage (X)	150
Genome completeness (%)	98.79
Contamination (%)	1.15
Number of contigs	124
Number of contigs (≥ 1000 bp)	124
Longest contig size (bp)	448,142
Shortest contig size (bp)	1145
N50	126,162
N90	30,136
L50	17
L90	54
Genes (total)	5794
CDSs (with protein)	5673
CDSs (without protein)	61
tRNA	53
rRNA	1, 1, 1 (5S, 16S, 23S)
ncRNA	4

**Fig. 3:**
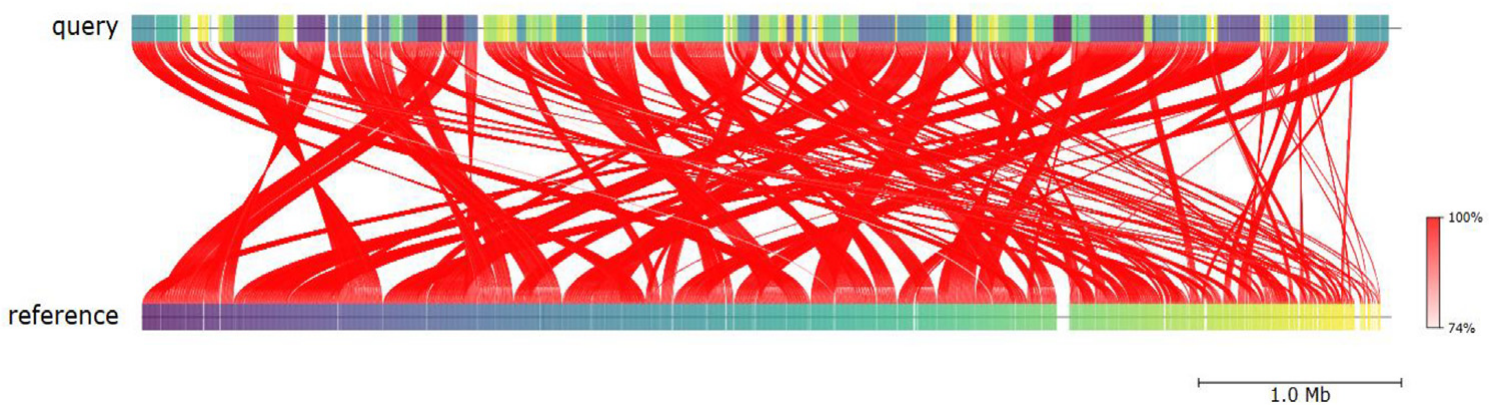
Average nucleotide identity (ANI) showing the similarity between *P. aeruginosa* PA-Mosul-25 (query genome) and *P. aeruginosa* WH-SGI-V-07,060 (reference genome).

Comparative genome analysis demonstrated that strain PA-Mosul-25 is most closely related to *Pseudomonas aeruginosa* strain WH-SGI-V-07,060 (NCBI RefSeq: GCF_001449145.1), a hospital-associated isolate from the United States exhibiting a multidrug-resistant (MDR) profile against several β-lactams and other antibiotic classes, supported by a Z-score of 0.99991, a digital DNA-DNA hybridization (dDDH) value of 90.9 % and an average nucleotide identity (ANI) of 98.8 % (Fig 3.). Multilocus sequence typing (MLST) analysis based on seven housekeeping loci (Table 2) classified strain PA-Mosul-25 as sequence type ST357.

**Table 2:** MLST profile of *P. aeruginosa* PA-Mosul-25, assigned to sequence type ST357 based on the PubMLST database*.*

Locus	Identity	Coverage	Length	Gaps	Allele
*acsA*	100	100	390	0	*acsA_*2
*aroE*	100	100	498	0	*aroE_*4
*guaA*	100	100	373	0	*guaA_*5
*mutL*	100	100	442	0	*mutL_*3
*nuoD*	100	100	366	0	*nuoD_*1
*ppsA*	100	100	370	0	*ppsA_*6
*trpE*	100	100	443	0	*trpE_*11

The antimicrobial susceptibility profile of *P. aeruginosa* PA-Mosul-25 as determined by VITEK 2 system is presented in Table 3.

**Table 3:** Antimicrobial susceptibility profile of *P. aeruginosa* PA-Mosul-25 including MIC values and categorical interpretations.

Antimicrobial	MIC (µg/mL)	Interpretation
Piperacillin/tazobactam	32	I
Cefazolin	≥ 64	R
Ceftazidime	8	S
Cefepime	16	I
Imipenem	0.5	S
Meropenem	≤ 0.25	S
Amikacin	≥ 64	R
Gentamicin	≥ 16	R
Ciprofloxacin	≥ 4	R

Genome screening revealed the presence of four β-lactam–associated resistance genes (*bla*_VEB-9_, *bla*_OXA-10_, *bla*_OXA-50_, and *bla*_PAO_). In addition, nine other antimicrobial resistance genes were detected (*aac*(6′)-Il, *aad*A1, *sul*1, *dfr*B2, *fos*A, *tet*(A), *cat*B7, *Oqx*B, and *qac*E), indicating potential resistance to multiple antibiotic classes (Table 4).

**Table 4:** Antibiotic resistance genes identified in the genome of *P. aeruginosa* PA-Mosul-25 using the CARD v4.0.1 and ResFinder v4.7.2 databases.

Resistance gene	Drug class	Resistance mechanism	Predicted phenotype	Identity (%)	PMID
*bla*_VEB-9_	beta-lactam	antibiotic inactivation	cefoxitin, ceftazidime, piperacillin, piperacillin+tazobactam, aztreonam, ticarcillin, ticarcillin+clavulanic acid	100	21,149,627
*bla*_OXA-10_	beta-lactam	antibiotic inactivation	amoxicillin, ampicillin, piperacillin, piperacillin+tazobactam, aztreonam	100	3126,705
*bla*_OXA-50_	beta-lactam	antibiotic inactivation	amoxicillin, ampicillin	99.5	15,155,197
*bla*_PAO_	beta-lactam	antibiotic inactivation	amoxicillin, ampicillin, cefepime, ceftazidime	99.37	19,258,272
*aac*(6′)-Il	aminoglycoside	antibiotic efflux	tobramycin, amikacin	100	7793,874
*aad*A1	aminoglycoside	antibiotic efflux	streptomycin, spectinomycin	99.75	23,934,739
*sul*1	folate pathway antagonist	antibiotic target replacement	sulfamethoxazole	100	Unpublished
*dfr*B2	folate pathway antagonist	antibiotic target replacement	trimethoprim	100	Unpublished
*fos*A	fosfomycin	antibiotic inactivation	fosfomycin	100	unpublished
*tet*(A)	tetracycline	antibiotic efflux	tetracycline, doxycycline	99.6	12,888,597
*cat*B7	amphenicol	antibiotic inactivation	chloramphenicol	98.59	10,361,706
*Oqx*B	quinolone	antibiotic efflux	ciprofloxacin, nalidixic acid	96.4	18,440,636
*qac*E	quaternary ammonium compound	antibiotic efflux	benzylkonium chloride, ethidium bromide, chlorhexidine, cetylpyridinium chloride	100	8494,372

The analysis of chromosomal point mutations within the quinolone resistance-determining regions (QRDRs) identified T83I substitution in the *gyr*A gene with high sequence identity (99.9 %), while S84L mutation was foundin the *par*C gene with identity sequence of 61 %. The analysis also identified two substitutions in the *gyr*B gene, namely V423F and I139R, which had identity sequence of 41 %.

Pathogenicity prediction analysis indicated a high probability of human pathogenic potential, with a mean score of 0.9902. The closest match in the pathogenicity prediction model was *Pseudomonas aeruginosa* strain PA155 (RefSeq: GCA_009299715.1), a human-derived isolate from Lebanon with a multidrug-resistance to β-lactams and fluoroquinolones, showing a Minkowski distance of 0.007378.

## 4 Experimental Design, Materials and Methods

*P. aeruginosa* PA-Mosul-25 was isolated from a swab sample collected from a floor surface corner in Al-Khansa Government Hospital. A sterile cotton swab (HiMedia, India) was used to sample approximately a 10 cm^2^ surface area by thoroughly wiping the surface. The swab was then used to inoculate Pseudomonas Agar Base (HiMedia, India) and incubated at 37 °C for 48 h. A presumptive colony showing growth on Pseudomonas Agar Base was subsequently subcultured on Nutrient Agar to obtain pure culture colonies. The isolate was then subjected to catalase and oxidase tests for primary identification. Final identification and antimicrobial susceptibility testing (AST) were performed using the VITEK 2 System v9.02.4 (BioMérieux, France) according to the manufacturer’s instructions. Minimum inhibitory concentration (MIC) values were interpreted in accordance with the Clinical and Laboratory Standards Institute (CLSI) guidelines (M100, 33rd edition, 2023) [7], and the results were interpreted as susceptible (S), intermediate (I), or resistant (R).

Genomic DNA (gDNA) was exstracted from fresh pure colonies of *P. aeruginosa* PA-Mosul-25 cultured on Nutrient Agar for 24 h at 37 °C using the Presto™ Mini gDNA Bacteria Kit (Geneaid, Taiwan) according to manufacturer’s instructions. The concentration and purity of the extracted gDNA were determined using a NanoDrop 2000 spectrophotometer (Thermo Scientific, USA). The genomic DNA library was constructed using the TruSeq Nano DNA Library kit (350 bp insert size), followed by whole-genome sequencing on the Illumina NovaSeq X platform with 2 × 150 bp paired-end reads at Macrogen Inc. (Seoul, South Korea). Raw sequencing reads were subjected to quality filtering and adapter trimming using Trimmomatic v0.30 with default parameters [8]. The high-quality reads were assembled de novo using SPAdes v4.2 [9] applying k-mer lengths of 21, 33, 55, and 77.

Genome annotation was performed using both the NCBI Prokaryotic Genome Annotation Pipeline (PGAP) v6.10 [10] and the Rapid Annotation using Subsystems Technology (RAST) server [11]. For RAST annotation, the following parameters were applied: taxonomy identifier 287 (*Pseudomonas aeruginosa*), domain Bacteria, genetic code 11, and the RASTtk annotation. Genome assembly statistics were generated using QUAST v5.3.0 [12], while genome completeness and contamination were evaluated using CheckM v1.2.4 [13].

Comparative genome analysis between *P. aeruginosa* PA-Mosul-25 and *P. aeruginosa* WH-SGI-V-07,060 was performed by calculating the Z-score using JSpecies v5.0.3 [14]. Digital DNA–DNA hybridization (dDDH) values were estimated using the Genome-to-Genome Distance Calculator (GGDC) v3.0 web server [15]. Average nucleotide identity (ANI) analysis was conducted using the Proksee server [16]. Multilocus sequence typing (MLST) and allele profiling were carried out using the PubMLST database [17].

Antimicrobial resistance genes and chromosomal point mutations were predicted using both the Comprehensive Antibiotic Resistance Database (CARD) v4.0.1 [18] and ResFinder v4.7.2 [19]. For CARD analysis, the parameters applied were: data type DNA sequence, criteria “perfect and strict hits only,” and sequence quality set to high quality/coverage. For ResFinder, a 90 % identity threshold, a minimum hit length of 80 %, and Pseudomonas aeruginosa as the reference species were used. The PathogenFinder v2 tool [20] was applied as a deep learning–based model to predict the potential pathogenicity of P. aeruginosa PA-Mosul-25 toward humans.

## Limitations

A limitation of this study is that the assembled genome remains incomplete and fragmented into 124 contigs, which may influence the overall accuracy and completeness of the genomic analyses*.*

## Ethics Statement

The bacterial isolate analyzed in this study was obtained from a hospital environmental sample and did not involve human participants, clinical specimens, or identifiable personal data. Therefore, ethical approval and informed consent were not required for this study*.*

## Credit Author Statement

**Hiba K. Saeed:** Conceptualization, methodology, investigation, validation, resources, data curation, writing; **Talal S. Salih:** Conceptualization, methodology, investigation, data curation, formal analysis, writing and editing.

## Data Availability

Pseudomonas aeruginosa strain PA-Mosul-25, whole genome shotgun sequencing project (Original data).
